# Quantifying Efficiency Gains of Innovative Designs of Two-Arm Vaccine Trials for COVID-19 Using an Epidemic Simulation Model

**DOI:** 10.1080/19466315.2021.1939774

**Published:** 2021-07-30

**Authors:** Rob Johnson, Chris Jackson, Anne Presanis, Sofia S. Villar, Daniela De Angelis

**Affiliations:** a Imperial College London, Department of Infectious Disease Epidemiology, London, UK; b MRC Biostatistics Unit, University of Cambridge, Cambridge, UK

**Keywords:** Adaptive design, Network model, Response-adaptive randomization

## Abstract

Clinical trials of a vaccine during an epidemic face particular challenges, such as the pressure to identify an effective vaccine quickly to control the epidemic, and the effect that time-space-varying infection incidence has on the power of a trial. We illustrate how the operating characteristics of different trial design elements maybe evaluated using a network epidemic and trial simulation model, based on COVID-19 and individually randomized two-arm trials with a binary outcome. We show that “ring” recruitment strategies, prioritizing participants at an imminent risk of infection, can result in substantial improvement in terms of power in the model we present. In addition, we introduce a novel method to make more efficient use of the data from the earliest cases of infection observed in the trial, whose infection may have been too early to be vaccine-preventable. Finally, we compare several methods of response-adaptive randomization (RAR), discussing their advantages and disadvantages in the context of our model and identifying particular adaptation strategies that preserve power and estimation properties, while slightly reducing the number of infections, given an effective vaccine.

## 1 Introduction

Vaccine trials are still in progress for SARS-CoV-2 and many vaccines are in development. So far, the trial designs proposed have been two-arm, individually randomized placebo-controlled trials, with the exception of the WHO R&D Blueprint (2020), which allows for new arms to be added. Vaccine trials in general have multiple, often competing, objectives which include establishing evidence on the efficacy of the vaccine, and conferring a health benefit to the trial participants as well as to the wider population (Bellan et al. 2017). Important decisions involved in designing any vaccine trial include the choice of trial population, whether randomization takes place at an individual or cluster level, the comparator, and the primary endpoint definition. When a vaccine trial is conducted in the midst of an epidemic, these decisions must address the specific challenges of: identifying an effective vaccine as quickly as possible to control the epidemic (Kahn et al. 2018); and the effect that variable infection incidence over space and time has on the power of a trial (Camacho et al. 2015). Efficiency gains that can address these challenges are a crucial topic of discussion, where “efficiency” includes: increasing power for a set number of participants or infections; reducing the required number of participants or infections for a given power; or reducing the time until conclusion for a given power. Kahn et al. (2018), Nason (2016) and Kahn et al. 2021) discussed such efficiency gains in the context of the three diverse trials designed for the 2014–2016 epidemic of Ebola virus disease (EVD) (Ebola ça Suffit Ring Vaccination Trial Consortium 2015; Kennedy et al. 2016; Widdowson et al. 2016).

In this article, we focus on three elements of trial design for testing vaccines in an epidemic that might improve power and efficiency: recruiting participants at the highest risk of infection; making more efficient use of the data on participants who are infected earliest in the trial; and RAR.

Recruitment of participants at highest risk of infection has been suggested (Kahn et al. 2018; Nason 2016; Kahn et al. 2021) to be more efficient than random recruitment. If high-risk individuals can be confidently identified, then recruiting from them can increase the number of cases that are observable in a fixed window of time. Power is not only directly increased by increasing the total number of events observed, but also indirectly by reducing the risks of the incidence rate considerably changing during the trial. “Ring” recruitment, where contacts of known cases are recruited, was implemented in a vaccine trial for EVD (Henao-Restrepo et al. 2017). In the ongoing COVID-19 pandemic, many nations developed contact tracing systems to contain the spread of disease (European Centre for Disease Prevention and Control 2020), and contact tracing has been explored as a means for rolling out a tested vaccine (MacIntyre, Costantino, and Trent 2020), but, to the best of our knowledge, contact tracing systems have not been formally used to define a recruitment strategy for vaccine trials. Such a strategy to recruit patients for an ongoing treatment trial has been adopted to some success (Cake et al. 2021).

An important disadvantage of recruiting people at risk of imminent infection is that by the time patients are randomized, infection may already have occurred and thus cannot be prevented by the trial vaccine. Furthermore, even if infection has not occurred at randomization, it might still occur before the vaccine induces a protective effect. Including such individuals in a conventional analysis of the trial data would lead to underestimation of the vaccine efficacy (VE). Alternatively, participants observed to be infected before a particular time would commonly be excluded (as in e.g., Henao-Restrepo et al. 2017). However, since the time from (unobserved) infection to symptoms is variable, later cut-off times may exclude people who should have been included, thus reducing power, while earlier cut-off times will lead to underestimation of efficacy. Therefore, a more efficient way of using this information is desirable when ring recruitment is used.

Some adaptive designs may improve efficiency, confer health benefits to trial participants, or in some cases achieve both(Pallmann et al. 2018; Burnett et al. 2020), including in the context of COVID-19 treatment trials (Stallard et al. 2020) and of vaccine trials (Kahn et al. 2021). RAR, in which the proportion of people randomized to a particular trial arm is modified based on accumulated data observed at an interim analysis, has been suggested (Kahn et al. 2021; Scott 2020) to have the potential to balance the competing objectives of health benefits to the trial participants, power and time until a conclusion is reached. Brueckner et al. (2018) compared different RAR designs for trials of treatments during an epidemic. However, the performance of RAR has not been specifically quantified in the context of vaccine trials and its use in this context still remains debated (Proschan and Evans 2020; Villar, Robertson, and Rosenberger 2020).

We present an epidemic simulation study to assess the impact on operating characteristics of the three proposed design elements in a specific plausible COVID-19-like situation. This study also serves as a methodological example of how epidemic simulation can be used to evaluate trial designs in any emerging epidemic, subject to the development of models for the specific pathogen, epidemic and social context.

First, we assess ring-type designs that recruit contacts of infected people in the context of individual randomization. Second, we develop and evaluate a novel method to avoid excluding all data from early infections, using weights that are estimates of the probability that a person was infected after vaccine-induced antibody response. Finally, we compare various RAR procedures, under ring recruitment and downweighting early cases. We evaluate several operating characteristics of four different frequentist and Bayesian methods for updating allocation probabilities.

In Section 2, we describe the network epidemic transmission model, the common characteristics of the trials we simulate, and the details of the design choices that we compare. In Section 3, we present the estimated operating characteristics of each design. In Section 4, we conclude with a discussion of our findings, their limitations, and the potential for further work. Full details of the simulation model and trial mechanics are described in the online supplement (OS) Section A, further details of the analysis method in OS Section B, supplementary results in OS Section C, and code is available at *github.com/robj411/ADAGIO/COVID19*.

## 2 Simulation Study

### 2.1 Network Epidemic Model

We use a network model to simulate an epidemic occurring in a population in which the vaccine trial operates. There are two components to the model, following Hitchings et al. (2018): the network that describes relationships between individuals, and the transmission model that describes the dynamics of disease via these relationships. Both the disease transmission network and the network for tracing contacts of identified cases are subnetworks of the relationship network.

#### 2.1.1 Relationship Network

Our network is an undirected graph with vertices, or nodes, representing the *N_I_* individuals in the population of interest, and edges representing connections, or relationships, between individuals. These include relationships between people who know each other, defining the contact networks of the individuals (“known contacts”), and random relationships, between people who encounter each other only transiently (“transient contacts”). Transient contacts might include, for example, encounters between people who are traveling or in supermarkets, and are not defined to be part of contact networks.

We consider three types of relationships: within household; in workplaces; and transient. Together, the household and the workplace edges are the *known* edges that make up the known contacts. We assign a “relationship weight” of 0.1 to transient connections, compared to relationship weight 1 for non-transient relationships, to reflect the smaller probability of contact sufficient to enable transmission between transient connections than between acquaintances.

Each individual has a set of attributes such as household and age (< 19, 19–65, > 65). Every individual in a household is connected to every other individual in the household. The age and household size distribution is taken from the United Kingdom 2011 Census (Office for National Statistics 2011). People aged 19–65, and one fifth of people aged 65+, are connected to 15 other people, on average, via a workplace. The number of people in the workplace is reflective of the likely number of people with whom an infrastructure is shared, rather than the number of colleagues. Individuals within a workplace are completely connected to one another.

**Figure 1:**
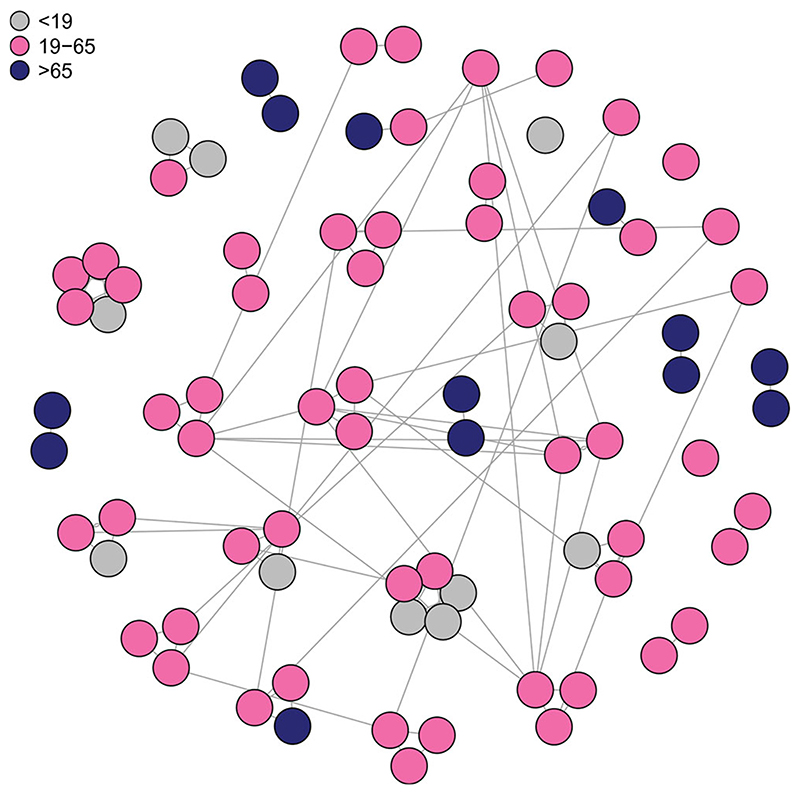
Example relationship network showing 80 people. “Known” edges between housemates and colleagues are shown. Individuals are colored by age group and clustered into households.

Finally, random edges are added, to allow around ten transient connections per person, that is, potential transmission encounters that would not be recalled or anticipated through contact tracing. The result is an average of 20 connections per person, of which ten have a weight of 1 and 10 have a weight of 0.1. An example is shown in Figure 1. Full details of the network are given in OS Section A.

#### 2.1.2 Disease and Trial State Transitions

**Figure 2:**
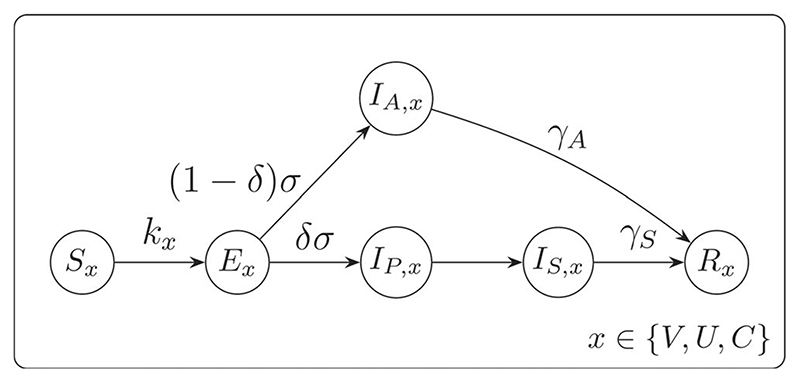
Disease-state transition model for members of the population who are not enrolled (*U*) and those enrolled and vaccinated (*V*) and those enrolled to the control group (*C*). Arrows show possible transitions between states, labeled by the rates.

Individuals’ disease states and possible transitions are described by a compartmental SEIR model (Figure 2), with a structure similar to Camacho et al. (2015) and Danon et al. (2020). The possible states are *S* Susceptible, *E* Exposed (infected but not infectious), *I_A_* Infectious and asymptomatic, *I_P_* Infectious and pre-symptomatic, *I_S_* Infectious and symptomatic and *R* Removed. Trial enrollment states are represented by the subscript *x*, where *x* = *U* is not enrolled, *x* = *V* is enrolled and vaccinated, *x* = *C* is enrolled and in the control arm.

Every individual *i* starts susceptible and unenrolled, in state *S_U_*, except the index case who starts in *E_U_*. The infection hazard *k_x_* (*i*) for an individual *i* in susceptible state *S_x_* is a function of the per-contact transmission rate (see OS Section A), their contact network, their vaccination status *x_i_* and the VE 0 ≤ η ≤ 1, defined as the percent reduction in attack rate for vaccinated people compared to unvaccinated people, assuming the vaccinated population have reached the maximum state of protection they are capable to reach (Weinberg and Szilagyi 2010; Shim and Galvani 2012). We equate this state with vaccine-induced antibody response (Hudgens, Gilbert, and Self 2004). An infected individual in the exposed state *E_x_* becomes infectious but asymptomatic with probability 1 − δ, moving to state *I_A_* at rate (1 − δ)σ. The remaining proportion δ becomes infectious and pre-symptomatic, moving to state *I_P_* at rate *δσ*. The transition rate σ corresponds to an incubation period ξ ∼ 2 + Γ(shape = 13.3, rate = 4.16) (Li et al. 2020) and we set δ = 0.8 (Buitrago-Garcia et al. 2020).

The asymptomatic individuals remain asymptomatic and therefore stay in *I_A_* for their whole infectious period, *ψ* ∼ 1 + Γ(shape = 1.43,rate = 0.549) (Li et al. 2020), corresponding to a rate *γ_A_*, before moving to the removed state *R*. The pre-symptomatic individuals in *I_P_* move to *I_S_*, after a deterministic time of 1 day (Kucharski et al. 2020). Symptomatic individuals remain in *IS* for the remainder of their infectious period, ψ − 1 ~ Γ(shape = 1.43, rate = 0.549), corresponding to rate *γ_S_*, before moving to the removed state *R*. For simplicity, we assume that symptomatic persons do not leave their homes, so that an infectious person in *I_A_* or *I_P_* can infect their home contacts, their work contacts and their random contacts, but an infectious person in *I_S_* can only infect their home contacts. Transitions to state *R* imply removal from the infectious population: this can be due to death, hospitalization (and hence isolation), or recovery.

See OS Section A for full details of the transmission model.

We simulate 500 households, corresponding to populations of around 1000 individuals. Our simulated trials operate on a time unit of one day, and have total duration of the order of 100 days. We assume that one contact network (corresponding to one index case) is initiated every day. Initiation is the moment where all nodes in the network are in state *S_U_* except one individual who is on their first day in state *E_U_*. Enrollment can only begin when this individual reaches state *I_S_*. Each trial participant who is enrolled has as their reference day the day on which they were enrolled. We simulate a VE of either no effect *η* = 0 or a positive effect *η* = 0.7.

### 2.2 Trial Design

The elements of trial design we explore are specific to an infectious disease with the dynamics and means of spread of EVD or COVID-19 (i.e., through close person-to-person contact) with the primary endpoint defined as a reverse transcriptase polymerase chain reaction (RT-PCR)-confirmed diagnosis of current symptomatic infection. In addition, our trial is for a single-dose vaccine whose time to development of antibodies is fast, that is, a (hypothetical) disease-specific antibody test would go from negative to positive within 15 days, for all participants with high confidence (Marzi et al. 2015; Poland, Ovsyannikova, and Kennedy 2020). We use antibody response as a proxy for a vaccine-induced protective immune response in our simulations.

We develop a trial design step by step. First, we design a “base case.” Then, we choose one element of the design to change(e.g., the recruitment strategy from random to “ring”) and compare the new design to the original. We then take forward the betterperforming design and make a comparison on another element of the design. In the starting “base case” for our simulations, participants are recruited at random; the allocation probabilities are fixed and equal (fixed randomization, FR); the final followup time is 25 days after randomization, similar to the follow-up time of 21 days used for EVD (Hitchings, Grais, and Lipsitch 2017; Ebola ça Suffit Ring Vaccination Trial Consortium 2015); the trial terminates once 24 confirmed cases have been observed (determined to achieve 80% power for a VE of 0.7), and is analyzed assuming everyone receives the intervention they were randomized to.

In the base case, similar to Henao-Restrepo et al. (2017), who excluded those exhibiting symptoms within 10 days in their trial for an EVD vaccine, and WHO R&D Blueprint (2020), who propose 14 days for COVID-19, we exclude cases that display symptoms within nine days of randomization. Our rationale for 9 days aligns with our assumed time to vaccine-induced antibody response and the estimated incubation period for SARS-CoV-2 (Li et al. 2020). Notice that the Blueprint uses a time-to-event outcome, whereas we use a binary outcome (PCR-confirmed diagnosis of COVID-19), which enables us to use well-known methods of RAR, and for which we have a maximum follow-up time of 25 days.

The primary endpoint in our simulations corresponds to a disease endpoint, where any person who becomes symptomatic is PCR tested and diagnosed. We assume that through surveillance and self-reporting, symptomatic participants correspond to confirmed cases, that is, all symptomatic people report their symptoms and onset date accurately, all tests have perfect accuracy, and each symptomatic person’s positive result is available by the time of the end of their follow-up period of 25 days. In reality, some people test positive for SARS-CoV-2 but show no symptoms. In our simulations, these people are infectious but not symptomatic. In terms of the network epidemic model, they continue to behave as if they are not infectious, so they retain contacts whom they can infect over their infectious period. In terms of the trial, they are not diseased and so are counted in the group of trial successes. An “infection endpoint” (Hudgens, Gilbert, and Self 2004), where only an “uninfected” outcome is a success of the vaccine, would require routinely testing all participants.

We use an event-driven approach (Schoenfeld 1983)for trial size determination. The trial is terminated following the accumulation of a prespecified number of confirmed cases, rather than a prespecified number of enrolled participants (which corresponds to a fixed sample size). In the comparisons we make, all designs employ randomization at the individual level and compare to a placebo control, as suggested by Kahn et al. (2021).

### 2.3 Recruitment

We compare random recruitment to the ring-recruitment strategy employed in Henao-Restrepo et al. (2017). Participants are eligible for enrollment when someone in their contact network is confirmed as a “case.” We use “ring” with reference to the method for recruitment, and we use individual-level randomization, whereas other implementations of the strategy have used cluster randomization (Henao-Restrepo et al. 2015). In cluster randomization an individual is eligible only if the contact network of its index case is eligible, based on the trial’s inclusion criteria. Therefore, individual randomization can enroll more participants per index case as eligibility is individual. We define the ring as consisting of contacts and contacts of contacts. These contacts are found through contact tracing, as described by the European Centre for Disease Prevention and Control (2020). In OS Section C.1, we show how the success of the ring recruitment method depends on the ability of contact tracing to identify those at imminent risk of infection.

### 2.4 Weighted Exclusion

Explicit mention of time to vaccine-induced antibody response is not often made in the definition of VE, although exclusion rules often make reference to this time: participants who are confirmed as cases within a certain number of days of randomization are excluded from the analysis (“binary exclusion”), as they are assumed to have been infected either before randomization or before the vaccine has a chance to take effect (Dean et al. 2019; Henao-Restrepo et al. 2017).

However, by excluding observations, we lose some information. Our ideal endpoint is whether or not a participant became infected after randomization and vaccine-induced immune response, where participants who became infected before are excluded from the trial. We typically do not know the day on which a vaccinated person develops infection- or vaccine-induced antibodies. However, we can observe whether or not a person is symptomatic, at which point infection can be confirmed through laboratory testing. Therefore, for the analysis at the end of the trial, we propose supplementing the primary endpoint—the observed infection status at day 25—with a retrospective exclusion criterion: we use our knowledge of the disease (e.g., the incubation period) and the day a person becomes symptomatic to weight their inclusion in the analysis.

We define our primary endpoint for the final analysis as PCR-confirmed, symptomatic infection and, in addition, the day of symptom onset relative to the day of randomization, which informs a retrospective exclusion criterion. This exclusion of participants is expressed as an “inclusion weight” between 0 and 1, computed at each round of analysis, and applied retrospectively, as if we had excluded a participant from the beginning (“continuous inclusion”). When confirmed cases are weighted, the number of cases becomes the *effective number of cases*, and the sample size the *effective sample size*.

The probability that a person whose symptoms began after vaccination was infected after vaccine-induced antibody response depends on the effect of the vaccine. If the vaccine is effective, then this probability is smaller than it would be if the vaccine had no effect. Therefore, as an additional development, we estimate the VE and the inclusion weights together iteratively, so that both would be updated if (as in a response-adaptive design) we were to recalculate them as results accumulate. See OS Figure 1 for an illustration of how the weights are obtained, and for the mathematical derivation. We assess this weighting method in terms of power and Type I error compared to binary exclusion.

### 2.5 RAR

Let π_*v*_ be the probability of being allocated to arm *v*, where *v* is 0 for control and 1 for experimental. Thus far we have considered fixed-randomization (FR) designs, in which the allocation probabilities are fixed and equal (π_0_ = π_1_ = 0.5) throughout the trial. In RAR, allocation probabilities are updated at prespecified moments in the trial using accumulated data according to a prespecified rule.

We set the frequency of adaptation to be every 25 days: all data accrued up to the adaptation day (up to the maximum follow-up time of 25 days post randomization) are used to generate the probabilities. Therefore, the allocation probabilities are updated after groups of participants of random size, rather than individuals, and the first group acts as a “burn-in phase” where there is an equal probability of receiving each arm. We present frequentist and Bayesian methods to generate π1.The methods all require first estimating *p*_0_ and *p*_1_: the probability of being uninfected up to the maximum follow-up time for the control and experimental arms, respectively. We denote the estimates ${\hat{p}}_{0}$ and ${\hat{p}}_{1}$ .

The probabilities are estimated as the number of successes over the total number of observations; ${\hat{p}}_{v}=1-f_{v}/N_{v}$ , where *f_v_* is the effective number of confirmed cases for arm *v*, and *N_v_* its effective sample size (Hu and Rosenberger 2006).

#### 2.5.1 Frequentist Response-Adaptive

In Rosenberger et al.’s (2001) method, favorable outcomes are optimized subject to a power constraint, so that $\rho_{\text{Ros}}=\sqrt{\frac{{\hat{p}}_{1}}{{\hat{p}}_{0}}}$ is the optimal randomization ratio of experimental to control. Then the allocation probability to the experimental arm is

$$\pi_{1}=\frac{\rho_{\text{Ros}}}{\rho_{\text{Ros}}+1}=\frac{\sqrt{{\hat{p}}_{1}}}{\sqrt{{\hat{p}}_{1}}+\sqrt{{\hat{p}}_{0}}}.$$

We also consider the Neyman method, which is designed to maximize power (Rosenberger et al. 2001), by setting

$$\pi_{1}=\frac{\sqrt{{\hat{p}}_{1}\left(1-{\hat{p}}_{1}\right)}}{\sqrt{{\hat{p}}_{0}\left(1-{\hat{p}}_{0}\right)}+\sqrt{{\hat{p}}_{1}\left(1-{\hat{p}}_{1}\right)}}.$$

For example, if the infection rate for the control arm is 80% ( ${\hat{p}}_{0}=0.2$ ) and that in the experimental arm is 20% ( ${\hat{p}}_{1}=0.8$ ), then the Rosenberger et al. method would allocate participants in a 2:1 ratio favoring the experimental arm ( $\rho_{\text{Ros}}=\sqrt{4}=2$ , and π_1_ = 2/3). The Neyman rule has π_1_ = 0.5 (and *ρ*_Ney_ = 1). For these methods, in the case that ${\hat{p}}_{0}\left(1-{\hat{p}}_{0}\right)=0$ or ${\hat{p}}_{1}(1-{\hat{p}}_{1})=0$ , we set π_0_ = π_1_ = 0.5.

#### 2.5.2 Bayesian Response-Adaptive (Thompson Sampling)

The Bayesian methods define the allocation probability in terms oftheposteriordistributionsof*p_i_* given a uniform prior and the observed data, Beta(1 + *N_v_* — *f_v_*, 1 + *f_v_*). Then *π_i_* is estimated by sampling as (Thall and Wathen 2007)

(1) $$\pi_{1}=\frac{\text{Pr}{\left(p_{1}>p_{0}\right)}^{\phi }}{\text{Pr}{\left(p_{1}>p_{0}\right)}^{\phi }+\text{Pr}{\left(p_{1}<p_{0}\right)}^{\phi }},$$

where we define a tuning parameter *φ*, which tempers the speed with which the allocation probability can reach extreme values (0 or 1). For Thompson sampling (TS), we set *φ* = 1, so π_1_ is just Pr(*p*_1_ > *p*_0_), and for TS with tuning (TST), *φ* = *j*/*e*, with *j* the day of the current update, and *e* the trial’s expected total duration. One might instead choose to adapt according to number of cases seen, so that *e* = 24 effective cases. *φ* therefore takes the value 0 at the beginning of the trial and goes to 1 as the trial progresses.

The Thompson sampling algorithm has a possibility of generating extreme allocation probabilities. While tuning limits this, we find that the TST method still tends to 1 over few adaptations. We therefore set limits to the allocation probability: we use a value of 0.8 if Equation (1) returns an allocation probability above 0.8, and we use a value of 0.2 if Equation (1) returns an allocation probability below 0.2 for both implementations of Bayesian RAR. We additionally terminate the trial early and conclude efficacy if Equation (1) returns a value of 0.99 (Brueckner et al. 2018).

#### 2.5.3 Time Trends

The epidemic unfolding in real time can give rise to temporal trends in incidence of the disease among participants, also referred to as “patient drift” (Proschan and Evans 2020; Villar, Robertson, and Rosenberger 2020). Patient drift affects all arms in the same way, and might be induced by a natural increase or decrease in incidence, or a step change due to government policy on social contact, or a change in the recruitment process. As both the adaptive trial design and the epidemic change over time, we must account for time dependencies of disease exposure when inferring the effect of the experimental vaccine.

Here, we use randomization-based inference as described by Simon and Simon (2011): we resample the data in order to generate a new null distribution for the test statistic to which to compare the one we compute. We present the resulting power and Type I error rates alongside the uncorrected values from standard testing.

### 2.6 Evaluation

We simulate *N_T_* trials, where each “trial” involves independent networks—as many as are required to achieve a particular total effective number of cases. The null hypothesis is no effect of the vaccine, *H*_0_ : η = 0 and the alternative hypothesis is a positive VE, *H*_1_ : η = η_1_ for a certain η_1_ > 0.

We report operating characteristics including the number of people enrolled and the number of confirmed cases, the power, the estimated VE, and the Type I error rate, alongside the details of the design. The duration of the trial is reported in days, and an average of *N_P_* participants are enrolled per day, according to the properties of our simulated network and enrollment rate.

The VE, 0 ≤ η ≤ 1 is estimated as

$$\hat{\eta }=1-\frac{f_{1}}{N_{1}}/\frac{f_{0}}{N_{0}},$$

where *f_v_*, *v* = 0, 1 is the effective number of cases in arm *v* and *N_v_* the effective number of participants in arm *v*. The VE is estimated using all simulations under the positive effect, whether or not the trial realization concluded efficacy.

Power is the probability of correctly rejecting the null hypothesis *H*_0_, and is estimated as the proportion of simulations under the alternative *H*_1_ for which the *H*_0_ is rejected. Type I error rate is the probability of incorrectly rejecting *H*_0_ when it is true, and is estimated as the proportion of simulations under the null for which the null hypothesis was rejected (see OS Section B for details). Other results presented (e.g., the numbers of people enrolled and confirmed as cases) are computed under the alternative unless stated otherwise.

Additionally, we report a novel metric to evaluate the different vaccine trial designs: the “prevented exported infections.” It is defined as the reduction in expected number of infection events of people not in the index case’s contact network, for 100 contact networks, comparing a trial realization with no vaccine effect with one with a positive effect of *η*_1_, in the case that the vaccine prevents infection as well as disease. While we do not expect this metric to be predictive of actual numbers of infections occurring, the relative numbers between methods are indicative of the trials’ possible or probable effects on the wider epidemic.

## 3 Results

The results in this section are from simulations of *N_T_* = 10,000 trials, where the alternative hypothesis positive effect is set to *η*_1_ = 0.7. We assume one contact network is enrolled per day. Given the network sizes and enrollment rate we assume, an average of *N_P_* = 32 people are enrolled per day.

### 3.1 Recruitment

Where recruitment is random, rather than through contact tracing, we have to recruit many more participants (Table 1), and many more people in the general population need to become infected,^1^ in order for the requisite number of infections to be observed among those recruited. In addition, fewer exported infection events are prevented. We carry forwards the ringrecruitment design as the “base case” for further comparisons.

### 3.2 Weighted Exclusion

In Table 2, we show results that suggest that by downweighting (“Continuous”) inclusion, rather than applying a binary rule, there is an increase in power of 0.07, and the VE estimate is closer to the true value of 0.7. The gain in power is in large part due to accounting for the VE when determining which early cases are likely to have been infected before randomization. Other operating characteristics are similar between the methods.

### 3.3 RAR

The comparison between the FR trial design developed up to now and the suite of response-adaptive designs is shown in Table 3. We fixed the number of effective cases observed in the trial population to a total weight of 24 so that powers were comparable, which sets the health cost to the trial participants for the Neyman, Rosenberger et al. and FR methods. We can then trade off the power against time to conclude. The Neyman method, which by design maximizes power, in fact has much lower power once we correct for patient drift using a randomization based approach. The Rosenberger et al. method is most similar to the fixed and equal randomization design, matching it in terms of number of participants, participant allocation, Type I error and power, and number of participants vaccinated.

The Thompson sampling methods benefit from stopping early when efficacy can be concluded; the TS design is expected to be shortest among all designs (see OS Table 3 for operating characteristics when the trials do not terminate early). Thus, the health cost to the trial participants for these methods is not prespecified and the number of cases among participants must also be taken into account when evaluating the methods. The Thompson sampling methods (TST and TS) allocate more participants to an effective vaccine than control when it exists. As a result, there are fewer infections exported from the network, and the power is lower (both with and without correction). See OS Figure 2 for operating characteristics under different trends, and OS Figure 3 for trajectories of allocation probabilities.

Table 3 illustrates how adaptive designs can be compared and how one might choose a design given the current circumstances: that, at a cost of some power, a design can be chosen that will vaccinate more people, if the vaccine is effective. This might be preferable in circumstances where infection rates are high. On the other hand, where infection rates are declining, a trial that maximizes power might be preferable, since it would be more challenging to observe cases quickly. Such a design likely would not prioritize vaccination, prioritizing instead information gain in order to increase the chance of identifying an efficacious vaccine. In OS Section C.5 we compare the same designs assuming instead that the trial must conclude within a certain number of days. Alternatively, designs could be compared in terms of the number of cases, the number of vaccinations, and duration, where all designs achieve the same power.

## 4 Discussion

Using simulation from a network epidemic model for COVID-19 with an embedded vaccine trial, we have illustrated the potential efficiency gains from three innovative two-arm trial design and analysis elements. These elements are designed to address the requirement in an epidemic to observe as many events as quickly as possible, both for control of the epidemic and information gain in the context of a highly variable and potentially low incidence. The utility of each of these elements will depend on the disease and context and should be assessed through simulation.

The first element, ring recruitment, which prioritizes individuals at imminent risk of infection, has been shown to substantially improve power and efficiency. Our proposed weighted analysis method makes more efficient use of the available data: reducing bias compared to fully including the data from people infected a short time after randomization, who the vaccine may not have had a chance to protect, while gaining power compared to completely excluding these data. While RAR may not offer a notably superior balance in terms of competing goals in the two-arm trials considered in this study, we nevertheless found that, given a moderately effective and safe vaccine, the adaptation method of Rosenberger et al. (2001) was comparable to a fixed randomized design, preserving Type I error and power while vaccinating slightly more people in the trial. Each of these three design elements could independently increase power, efficiency or patient benefit of a vaccine trial in particular contexts. Furthermore, the combination of all three simultaneously has the potential to improve a vaccine trial in an epidemic context both from the information gain and the health benefit perspective. We believe this conclusion is valuable given the limited scope for efficiency and participant benefit improvements that two-arm trials usually have.

Response-adaptive designs require an outcome that is observable soon after randomization, which can be achieved with a ring-recruitment strategy. Ring recruitment requires an efficient contact-tracing infrastructure to enable recruitment of participants at imminent risk of infection. Such contact tracing might be embedded in a national surveillance system aimed at containment, or might be part of the trial protocol. The ring design depends on the ability to anticipate among whom new infections will occur: specifically, if new cases occur among known contacts of cases, which might be ascertained through comparison of contacts traced and case registries. A COVID-19 treatment trial in the United Kingdom has successfully used the UK’s National Health Service contact tracing data to enhance their recruitment (Cake et al. 2021).

The success of the ring design depends also on the time taken to trace contacts relative to disease dynamics. Our simulations assumed that, on average, it takes ten days to identify and enroll a whole contact network, including the time for the index case to be confirmed (OS Table 1). The sooner participants are enrolled after their index case becomes infectious, the more chance there is for an efficacious vaccine to confer protection, as vaccination occurs earlier relative to the time that the participant is at risk. This timing will depend also on when infectiousness begins (which, for COVID-19, is before symptom presentation) and the disease’s incubation period. Fast enrollment relative to disease progression enhances information gain per participant as well as the potential health benefit to those in the experimental arm. If contacts cannot be traced fast enough, then ring recruitment would not be an appropriate method. Some debate about the limitations of the ring design of Henao-Restrepo et al. (2017) has been expressed, including the fact that it was a cluster randomized trial, and so “subject to the same biases as other cluster randomised trials” (Rid and Miller 2016). Here, instead, we have used individual randomization.

Most of the RAR designs we considered incurred a penalty in power. The penalty increased when we controlled for bias due to patient drift with rerandomization (Simon and Simon 2011). The more the allocation deviates from equality, the greater the design’s intended benefit but the larger penalty in terms of power. Bounding the allocation probabilities between 0.2 and 0.8 guards against very severe penalties, and would make the design more acceptable to stakeholders. We recommend that the tradeoffs between strictly preserving Type I error and the resulting power loss when using randomization based tests are considered carefully at design stage through extensive simulations. Alternative corrections, for example stratification (Chandereng and Chappell 2019), might prove less costly. The two-arm trials we considered give some insights into what a multi-arm response-adaptive design could offer. In a two-arm trial, power given a fixed number of participants can only be increased at the expense of participant benefit (Williamson et al. 2017; Villar, Bowden, and Wason 2015). In a multi-arm trial, worse-performing experimental arms can be deprioritized in favor of other arms (Tymofyeyev, Rosenberger, and Hu 2007). In our simulations, Type I error is not much inflated for the Thompson sampling methods since, under the null (i.e., no vaccine effect), 24 effective cases are typically observed soon after the end of the equal-randomization burn-in phase (OS Figure 3), so that the allocations are not very imbalanced. For a Thompson sampling design that adapts earlier relative to its endtime, we would expect to see an inflated Type I error, as in OS Figure 2.

We expect that the two-layer ring designs presented here would not suit a trial for a two-dose vaccine for COVID-19, since people at an imminent risk of infection are recruited, and so the majority of infections would occur before they could be prevented by a second dose. Thus, any estimates of efficacy would only describe the efficacy of the first dose. Adaptive designs may also be less appealing for a two-dose vaccine, since the disease outcome after two doses would take longer to observe.

In our simulations, we consistently underestimate the vaccine effect, and this is more pronounced for the ring recruitment designs. Our method of downweighting rather than excluding the earliest cases (which may not have been vaccine-preventable) is designed to improve power compared to excluding these cases, and controls bias compared to including them all. Any remaining bias could be controlled further by decreasing the weight assigned to early cases, at the cost of reducing power.

We have illustrated how simulation might be used to compare different designs and analysis options, in an approach similar to Hitchings et al. (2018). In practice, the network epidemic model must be specific to the particular setting, taking into account contact structures and governmental policies, as both network and epidemic dynamics will impact the trial designs’ operating characteristics. Through simulation, the design rules, such as the follow-up time and the requisite effective number of cases to achieve the desired power, can be established. Additionally, sensitivity to the structural and parametric assumptions underlying the network, epidemic, and trial models can be evaluated. To fully capture that the trial occurs within a real-life epidemic, the individual simulated contact networks could be embedded in a single, connected network on which the epidemic spreads, rather than simulated as independent units. Embedding the trial simulation more comprehensively in an epidemic model, where the trial participants from different contact networks interact with each other and where the trial can impact on the epidemic, would permit a formal quantification of the benefits and limitations of different design choices in different epidemic settings, as in Bellan et al. (2017). Such an analysis would enable a realistic assessment of the impact of more complex time trends at different stages of an epidemic, and of the potential impact on the epidemic of designs that vaccinate more people.

## Supplementary Material

Supplementary materials for this article are available online. Please go to www.tandfonline.com/r/SBR.

Supplementary File

## Figures and Tables

**Table 1:** Comparison of designs where participants are recruited following the ring strategy vs. recruited at random.

Recruitment	Number of participants	Number of confirmed cases	Vaccinated	Power	Type I error	VE estimate	Prevented exported infections^1^
Random	11275 (2345)	34	5637	0.74	0.04	0.63 (0.18)	2.53
Ring	1929 (583)	45	965	0.73	0.04	0.58 (0.19)	6.29

NOTE: The trial follows the FR design with a follow-up time of 25 days. The trial ends when an effective number of 24 cases have been observed. Standard deviations for 10,000 simulations in brackets.

**Table 2:** Comparison of designs where the binary endpoint has a binary weight or a continuous weight.

Weighting	Number of participants	Number of confirmed cases	Vaccinated	Power	Type I error	VE estimate	Number of participants (null)	Prevented exported infections^1^
Binary	2083 (609)	49	1041	0.75	0.04	0.58 (0.18)	1302 (430)	6.25
Continuous	2136 (624)	50	1068	0.82	0.05	0.64 (0.19)	1277 (426)	6.35

NOTES: Participants are recruited following the ring strategy. The trial follows the FR design with a follow-up time of 25 days. The trial ends when an effective number of 24 cases have been observed for the continuous weight and 26 for the binary weight, in order to achieve comparable trial sizes in terms of the number of participants. Standard deviations for 10,000 simulations in brackets.

**Table 3:** Comparison of response-adaptive designs.

Adaptation	Number of participants	Duration(days)	Number of confirmed cases	Vaccinated	Power	Power(corrected)	Type I error	Type I error (corrected)	VE estimate	Prevented exported infections^1^
Ney	1947 (551)	85 (17)	54	816	0.83	**0.76**	0.06	**0.04**	0.67 (0.2)	4.62
Ros	2147 (630)	92 (20)	57	1083	**0.82**	0.79	**0.05**	0.04	0.64 (0.18)	5.38
TST	2032 (638)	88 (20)	51	1261	0.77	**0.76**	0.04	**0.04**	0.64 (0.19)	6.62
TS	1799 (740)	81 (23)	45	1148	0.80	**0.74**	0.04	**0.05**	0.67 (0.21)	5.99
FR	2137 (622)	91 (19)	57	1068	**0.82**		**0.05**		0.64 (0.19)	5.50

NOTES: The outcome has a continuous weighting. Participants are recruited following the ring strategy. The final follow-up time is 25 days. The trial ends when 24 effective cases have been observed. Standard deviations for 10,000 simulations in brackets. Correction for time trend uses the resampling method of Simon and Simon (2011). Bold type indicates the recommended method.

## Footnotes

1 ”Exported infections” is the expected number of infections of people outside the index case’s contact network, for 100 contact networks. “Prevented exported infections”is the difference between exported infections assuming a VE of 0.7 and a VE of 0 in the case that the vaccine prevents infection as well as disease.
