# Supplementary material for: Quantifying Efficiency Gains of Innovative Designs of Two-Arm Vaccine Trials for COVID-19 Using an Epidemic Simulation Model
Source: Stat Biopharm Res. Author manuscript; Available in PMC 2022 Jan 29. (PMC7612285; doi:10.1080/19466315.2021.1939774)
Supplement: Supplementary File [file EMS140631-supplement-Supplementary_File.pdf]

Quantifying efficiency gains of innovative designs of two-arm vaccine trials for COVID-19 using an epidemic simulation model: online supplement

Rob Johnson, Chris Jackson, Anne Presanis,
Sofia S. Villar, Daniela De Angelis

A. The COVID-19 model

Both the network model and the disease transmission model will be specific to a particular setting and, indeed, might change or become better informed over the course of the trial. Therefore, we use models without supposing that they will reflect the "truth" for any particular disease or scenario. Instead, we make choices we believe to be plausible in order to demonstrate the general methods, whose features will persist for settings such as these within the parameters we have described, i.e., diseases that pass from person to person, through contacts that can be traced.

A.1. Network model

A.1.1. Definitions

Our network is an undirected graph,

$$\mathcal{G} = (\mathcal{V}, \mathcal{E})$$

with vertices, or nodes,

$$\mathcal{V} = \{i, i = 1, ..., N_I\}$$

representing the N_I individuals, which we index with i, and edges

$$\mathcal{E} \subseteq \{\{i, j\} | (i, j) \in \mathcal{V}^2 \cap i \neq j\}$$

representing connections, or relationships, between individuals. Each node i has a set of attributes $\{a_1(i), a_2(i), ..., a_{N_a}(i)\}$.

The network operates at the level of the individuals. Each individual belongs to a household $\mathcal{H}_h$ and every individual in a household is connected to every other individual in the household, such that the induced subgraph $\mathcal{G}_1[\mathcal{H}_h]$ is completely connected:

$$\mathcal{E}_1[\mathcal{H}_h] = \{\{i, j\} | (i, j) \in \mathcal{V}[\mathcal{H}_h]^2 \cap i \neq j\}.$$

Let $\mathcal{H} = \{\mathcal{H}_h, h = 1, ..., N_H\}$ be the set of N_H households, which forms a partition of the vertices $\mathcal{V}$ (all subsets are mutually disjoint and their union is equal to the set). Let $a_1(i)$ be the household of individual i, i.e. $a_1(i) = h \Leftrightarrow i \in \mathcal{V}[\mathcal{H}_h]$. Then the size of a household is

$$\mathcal{O}(\mathcal{H}_h) = \sum_{i=1}^{N_I} \mathbf{1}_{a_1(i)=h}.$$

We start with $N_H = 500$ households with $\mathcal{O}(\mathcal{H}_h)$ people in each. The age and number distribution follows (Office for National Statistics, 2011). That is, each person has an age attribute,

$$a_2(i) \in \{1, 2, 3\}$$

where $a_2(i) = 1$ if person i is under 19, $a_2(i) = 2$ if person i is 19 to 65, and $a_2(i) = 3$ if person i is over 65.

We define an individual i to be part of the workforce via a "worker" variable $a_3(i)$, where

$$\begin{aligned} a_2(i) = 1 &\implies a_3(i) = 0, \\ a_2(i) = 2 &\implies a_3(i) = 1, \\ a_2(i) = 3 &\implies \begin{cases} a_3(i) = 0 & \text{with probability } 0.8 \\ a_3(i) = 1 & \text{with probability } 0.2. \end{cases} \end{aligned}$$

There are $A_3 = \sum_i \mathbf{1}_{a_3(i)=1} \approx 731$ people in the workforce. We define $A_3/15 \approx 49$ workplaces to which people with $a_3(i) = 1$ (that is, all those aged 19 to 65, and 1/5 of people over 65) are assigned following a multinomial distribution. We choose

to place on average 15 people in a "workplace", $\mathcal{W}_w$, which represents not their employment structure but a close shared use of the infrastructure. A useful guide might be how many toilets per person a place of work should have.

The workplaces are completely connected:

$$\mathcal{E}[\mathcal{W}_w] = \{\{i,j\}|(i,j) \in \mathcal{V}[\mathcal{W}_w]^2 \cap i \neq j\}.$$

Finally, 1000 totally random edges are added, which amounts to around ten per person:

$$\Pr\left(\{i,j\} \in \mathcal{E}\right) = \frac{10}{\left(\sum_h \mathcal{O}(\mathcal{H}_h)\right)}.$$

These edges correspond to potential transmission encounters that would not be recalled or anticipated through contact tracing. The result is an average of 20 connections per person, of which ten have a weight of 1 and ten have a weight of 0.1.

A.1.2. Parametrisation

The parameters we have used and their provenance are listed in Table 1.

Table 1: Parameters used in the COVID-19 disease transmission and vaccine trial model. $\mathcal{N}(l, \mu, \sigma)$ denotes a normal distribution truncated at l.

Parameter	Value	Source
Number of households	500	Chosen with reference to other choices
Household size	Draws from raw data	UK 2011 census
Workplace size	$\sim$ Poisson(15)	`https://www.hse.gov.uk/contact/faqs/toilets.htm`
β (per-contact infection or transmission rate)	0.01	Chosen with reference to other choices
Known edge weight (χ_h, χ_w)	1	Chosen with reference to other choices
Unknown edge weight (χ_n)	0.1	Chosen with reference to other choices
SARS-CoV-2 incubation period ξ	$\sim 2 + \Gamma(\text{shape} = 13.3, \text{rate} = 4.16)$	Li et al. (2020)
SARS-CoV-2 infectious period γ	$\sim 1 + \Gamma(\text{shape} = 1.43, \text{rate} = 0.549)$	Li et al. (2020)
Time to enrol whole contact network	$\sim \mathcal{N}(l = 0, 10.32, 4.79)$	Henao-Restrepo et al. (2017)
Time to vaccine-induced seroconversion	$\sim \Gamma(\text{shape} = 3, \text{rate} = 1)$	Plausibly all seroconverted within 14 days

A.2. Disease and trial state transitions

A.2.1. Disease transmission rules

A susceptible individual i belonging to arm x becomes "exposed" (i.e. transitions to state E) with rate

$$k_x(i) = \beta \left(\chi_h \mathcal{M}_i^{(h)} + \chi_h \mathcal{L}_i^{(h)} + \chi_w \mathcal{L}_i^{(w)} + \chi_n \mathcal{L}_i^{(n)} \right) (1 - \eta \cdot x_i), \quad x \in \{V, U, C\},$$

where h refers to household, w to workplace, and n to random. $\chi_h > 0$ is the edge weight for household contacts, $\chi_w > 0$ is the edge weight for workplace contacts, and $\chi_n > 0$ is the edge weight for random contacts. One could specify a different χ_h for pre- and post-symptomatic infectiousness, or even a changing profile over time, as described in He et al. (2020). $x_i = 1$ if person i is vaccinated and 0 otherwise. That is, we assume that the vaccine prevents infection, and does not change infectiousness or the likelihood of asymptomatic disease among those who become infected after vaccination or a prophylactic effect among those who were infected before. $\eta \leq 1$ is the vaccine efficacy, and $\beta > 0$ is the per-contact rate at which infectious people infect their susceptible contacts.[1] $\mathcal{M}_i^{(h)}$ is the number of I_S household (h) contacts of susceptible i.

$$\mathcal{L}_i^{(y)} = \sum_{j \in \mathcal{N}_{(y)}(i)} \mathbf{1}_{j \in \{I_{A,\cdot}, I_{P,\cdot}\}}, \quad y \in \{h, w, n\}$$

is similarly defined as the number of contacts who are infectious but not symptomatic.

Note that there is no infection other than from a contact (with the exception of the simulated "time trends", Figure 2). Thus any infection within a contact network came (directly or indirectly) from the contact network's index case.

Our model and parameter choices yield new infection events as occurring from pre-symptomatic people 58% of the time and from symptomatic people 42% of the time, omitting transmissions from people who never become symptomatic. In comparison to reported estimates (44% from 77 recorded transmission pairs (He et al., 2020), and 48 and 62% in Singapore and Tianjin, respectively (Tapiwa et al., 2020), we confirm that our simulation scenario is consistent with one with quick quarantine of close contacts (He et al., 2020).

[1] The unit of β is per contact per day; the edge weights χ are unitless. They can be thought to transform the static (or quenched) network into a dynamic or adaptive network through link deactivation (Kiss et al., 2017).

A.2.2. Trial rules

In our trial we define contact tracing as identifying only existing relationships ("acquaintances", or "those met before", in the terminology of Kucharski et al. (2020)) that might be, or might have been, a means for transmission. That is, a newly diagnosed person is asked to recall all of their contacts, and these individuals are contacted and asked likewise to list all their contacts. This makes our simulation and trial design most like the "self-isolation and manual contact tracing of acquaintances" of Kucharski et al. (2020). The relationships that are of interest will depend on the society and any concurrent actions, guidance or instruction from the state, which in our simulation include home and workplace contacts, while random contacts remain unknown. We assume unknown relationships are not recalled in contact tracing and, similarly, cannot be anticipated for recruitment of trial participants. We assume work and home relationships are recalled perfectly. By recruiting "contacts" and "contacts of contacts" into the trial, we are recruiting the people the index case lives with and their colleagues, and those the case works with and their housemates.

For our purposes, an index case for a contact network is a person identified as being in state $I_{S,\cdot}$ after the initiation of the trial. Eligible people are traced as described in European Centre for Disease Prevention and Control (2020) and those who consent are enrolled as soon as they are identified and give their consent. It takes on average ten days from symptom onset to enrol a contact network, which includes the time to report, the time to contact trace, and the time to enrol (Henao-Restrepo et al., 2017). Susceptible and exposed people are eligible for enrolment if they are a "known" contact of the index case and they are not already enrolled in the trial. Symptomatic $I_{S,U}$ people are excluded on the basis of their symptoms. R_U are excluded on the basis of their history, which we assume a perfect knowledge of in our simulation. This could result either from people being able to identify having had COVID-19, or from there being an accurate and reliable antibody test. Inclusion of R_U people in the simulated trial would result in a dilution of infections and would therefore require enrolment of more participants to maintain power. Their inclusion is advocated for reasons of safety testing.

The enrolment rate is $0 \leq \epsilon \leq 1$, which is the probability for each eligible person to enrol, where we use only contact structure and lack of symptoms to define eligibility. We use $\epsilon = 0.7$.[2] Enrolled participants are randomised to the experimental arm with probability $0 \leq \pi_1 \leq 1$, and $\pi_1 + \pi_0 = 1$. Enrolment following symp-

[2]Henao-Restrepo et al. (2017) report that 50% of people identified were eligible and enrolled, where their eligibility criteria excluded people who were pregnant, breastfeeding, or under the age of 18.

tom onset for the index case takes time α, which we describe following observed enrollment times (Henao-Restrepo et al., 2017). Alternatives include a Poisson distribution with parameter $\sim$ Uniform(1.5, 2.5) (Fyles et al., 2021). Ideally, this parameter would be determined according to observed contact-tracing efforts and the expected capacity of the trial team.

Those vaccinated have an additional wait time before reaching state S_V, which is development of immunity (or time to seroconversion or detectable antibodies), and which takes time τ. Transition from S_U to E_U is possible in this wait time. We assume that the vaccine effect before seroconversion is zero and that the vaccine effect after is the full effect of the vaccine.

Individuals who are in state E and are unenrolled are enrolled with the same probability (ϵ) as the susceptibles S_U, as they are asymptomatic. The same wait time for enrolment applies, but time to seroconversion does not, as the individual is already infected. If the participant transitions to $I_{S,U}$ before the recruitment time elapses, they will be excluded from the trial, as they will be showing symptoms and can be tested for confirmation.

Result accrual relies on surveillance and self reporting. For our simulations we assume that a fraction $1 - \delta = 0.2$ of infectious individuals are asymptomatic. These infection events go unreported in the trial results (but still contribute to onward transmission). Our simulation mirrors the rules given to the participants: that upon the onset of symptoms, they should stay at home, and notify the trial team, who will organise the PCR test. In this way, the day of symptom onset is recorded (to be confirmed by PCR). We assume that this information will be available at the end of the follow-up period (25 days). This means that we assume that a person who becomes symptomatic on day 25 is tested and confirmed on the same day. In our simulation we assume that the rules are followed without error.

We simulate one network at a time, beginning when the index case is identified. Transmission in each network is independent of all other networks in the trial. The networks are related only through the time reference, in that one contact network is initiated on each day.

Finally, we assume that randomisation, enrolment and vaccination all happen on a single day for each individual, although the day will differ between individuals.

B. Analyses, and exclusion criterion implemented at analysis points

Here we detail all equations to accompany Section 2.4, which describes the different ways we explore to analyse the outcome. We present the methods in the same order and use a single framework that describes all the methods in the same way.

B.1. Analysis of raw data

We have $j = 1, ..., N_I$ individuals. Each has a vaccination status, x_j, and a disease status y_j, where the vaccination status is dictated by the trial design and the disease status from the underlying epidemic model:

$$x_j = \begin{cases} 0 & \text{person } j \text{ not vaccinated} \\ 1 & \text{person } j \text{ vaccinated} \end{cases}$$

and

$$y_j = \begin{cases} 0 & \text{person } j \text{ not diagnosed} \\ 1 & \text{person } j \text{ diagnosed} \end{cases}$$

at the end of the trial.

The test statistic is a standard normal variable Z, where

$$Z = \frac{\hat{p}_1 - \hat{p}_0}{\sqrt{\sigma_0 + \sigma_1}}, \tag{1}$$

$$\hat{p}_v = \frac{\sum_{j:x_j=v,y_j=0} \omega_j}{N_v}, \tag{2}$$

$$N_v = \sum_{j:x_j=v} \omega_j \tag{3}$$

$$\omega_j = 1 \quad \forall j,$$

and

$$\sigma_v = \frac{\hat{p}_v(1 - \hat{p}_v)}{N_v}. \tag{4}$$

p_v is the true probability of not being a confirmed case if in arm v, and $\hat{p}_v$ is our estimate of it, defined as the proportion of people in arm v not confirmed, where $v = 0$ is the control arm and $v = 1$ is the experimental arm; N_v is the total number in arm v; and σ_v is the variance of the estimator $\hat{p}_v$. Power is defined as the proportion of Z values that exceed 1.64, which is the 95th quantile of a standard

normal distribution. ω_j is the weight of person j which, for the unweighted method, is 1 for all participants. In the descriptions that follow, we see that the weights ω define the retrospective exclusion criterion so that all methods use the same calculation and each is defined only by the definition of the weights.

B.2. Analysis using binary weighting

The binary-weighting method proceeds as above but considers also the day of commencement of symptoms, s_j. Individuals are excluded if $s_j < 9$ relative to a randomisation day of 0. We write this as the weight, ω_j, for each individual j, so that a weight of 0 equates to exclusion:

$$\omega_j = \begin{cases} 0 & s_j < 9 \\ 1 & s_j \geq 9 \text{ or } y_j = 0 \end{cases}$$

These are used together with Equations 1, 2, 3, and 4 as before.

B.3. Analysis using continuous weighting

Given person j's symptoms began on day s_j relative to their randomisation day of 0, τ and ξ are their unknown time to seroconversion[3] and incubation time, respectively. The probability they were infected after the trial began is $P(\tau + \xi < s_j)$. We assume τ and ξ are distributed between individuals as $\Gamma(\text{shape} = 3, \text{rate} = 1)$ and $2+\Gamma(\text{shape} = 13.3, \text{rate} = 4.16)$. Hence the distribution of $\tau+\xi$ is estimated by matching moments using the Welch–Satterthwaite equation, as described in Box (1954).

We estimate the vaccine efficacy $0 \leq \hat{\eta} \leq 1$ as

$$\hat{\eta} = 1 - \frac{f_1}{N_1} \Big/ \frac{f_0}{N_0},$$

where

$$\hat{f}_v = \sum_{j: x_j = v, y_j = 1} \omega_j .$$

[3]Note that we include time to seroconversion also for the control group, who don't receive the vaccine, and don't seroconvert.

Suppose from the cumulative distribution function of the gamma distribution we have a nominal probability, i.e. neglecting the effect of the vaccine, q_j that the day person j was infected, d_j, was after seroconversion on day D_c. We write the complement, the probability that person j was infected before day D_c, as $r_j = 1 - q_j$. We re-estimate their probability given that person j was vaccinated ($x_j = 1$):

$$\Pr(d_j > D_c | x_j = 1) = \frac{(1-\hat{\eta})x_j}{y_j + (1-\hat{\eta})x_j}, \tag{5}$$

because, if there is some efficacy, then they are more likely to have been infected before being vaccinated than after (relative to a vaccine that has no effect: $\Pr(d_j > D_c | x_j = 0) = q_j$). We solve this iteratively for $\hat{\eta}$ with reference to all observations j.[4] Then

$$\omega_j = \begin{cases} 1 & y_j = 0 \\ \Pr(d_j > D_c | x_j) & y_j = 1 \end{cases} \tag{6}$$

See Figure 1 for an illustration of the relationship between weight and vaccine effect. The resulting weights are used in Equations 1, 2, 3, and 4 as before.

Determining the inclusion weight for a vaccinated person

The inclusion weight for a vaccinated person j ($q_j = 1$), whose symptoms began after D_c (randomisation date plus an assumed time from vaccination to seroconversion) is the probability that they were infected after D_c. To determine this, first we make explicit the condition that their infection date d_j is less than their symptom date D_s. The conditional probability we want can then be decomposed into probabilities unconditional on the symptom date, as follows,

$$P(d_j > D_c | q_j = 1, d_j < D_s) = P(D_c < d_j < D_s, q_j = 1)/P(d_j < D_s, q_j = 1)$$

Then splitting the denominator into the probabilities of being infected in two different periods (before versus after D_c) gives

$$P(d_j > D_c | q_j = 1, d_j < D_s) = \frac{P(D_c < d_j < D_s | q_j = 1)}{P(d_j < D_c | q_j = 1) + P(D_c < d_j < D_s | q_j = 1)}$$

Since the probability of being infected before D_c doesn't depend on whether person j was vaccinated, $P(d_j < D_c | q_j = 1) = P(d_j < D_c | q_j = 0)$. We additionally

[4] This is solved as in expectation maximisation (EM). EM algorithms have been used and discussed in clinical trials for e.g. sample-size re-estimation (Gould and Shih, 1992; Friede and Kieser, 2002; Teel et al., 2015; Huang et al., 2018).

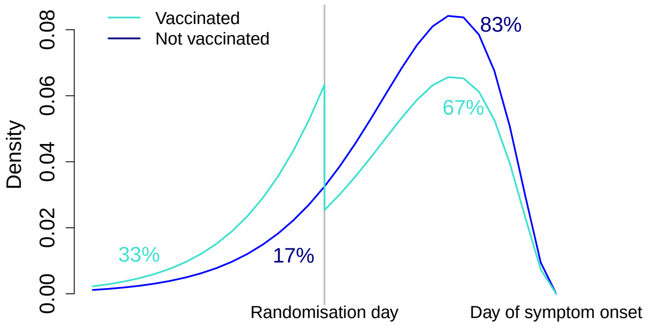

Figure 1: The probability that a person became infected on day x, given the day they showed symptoms, whether or not they were vaccinated, and the effect of the vaccine (where we omit vaccine-induced antibody response for simplicity of presentation). The inclusion weight given the day of symptom onset is the area under curve to the left of the randomisation day. The lines illustrate two participants who show symptoms on the same day. One (turquoise) is vaccinated; the other (navy) is not. Given the distribution of the incubation period, the navy person has a probability of 0.83 of having been infected after randomisation day. Their weight is therefore 0.83. Given that the vaccine efficacy estimate exceeds zero, the turquoise person is less than 83% likely to have been infected after randomisation day – 67%, for an estimated vaccine efficacy of 0.6. In the binary-weighting case, both these participants have weight 0 (if day of symptom onset is less than the threshold of nine days) or weight 1 (otherwise).

assume that

$$P(D_c < d_j < D_s | q_j = 1) = \psi P(D_c < d_j < D_s | q_j = 0)$$

where ψ is the relative risk of infection between a vaccinated and unvaccinated person, assumed to be constant through time, giving

$$P(d_j > D_c | q_j = 1, d_j < D_s) = \frac{\psi P(D_c < d_j < D_s | q_j = 0)}{P(d_j < D_c | q_j = 0) + \psi P(D_c < d_j < D_s | q_j = 0)}$$

Expressing the right hand side in terms of probabilities conditional on symptoms, by dividing the numerator and denominator by $P(d_j < D_s | q_j = 0)$, then gives the

weight for a vaccinated person j as

$$\omega_j = P(d_j > D_c | q_j = 1, d_j < D_s) = \frac{\psi \omega_j^{(0)}}{(1 - \omega_j^{(0)}) + \psi \omega_j^{(0)}}$$

where $\omega_j^{(0)} = P(D_c < d_j < D_s | d_j < D_s, q_j = 0) = P(D_c < d_j < D_s | q_j = 0)/P(d_j < D_s | q_j = 0)$ is the weight if person j were unvaccinated.

C. Supplementary results

C.1. Dependence of ring recruitment on contact tracing

As ring recruitment relies on contact tracing, here we present a sensitivity analysis that demonstrates how the success of the method depends on the "known fraction". We define the "known fraction" of a network as $r_s = e_s w_s/(e_s w_s + e_u w_u)$, the weighted sum of known edges over the total weighted sum of edges, where there are e_s known and e_u unknown edges, with relationship weights w_s and w_u respectively. The net result is that the known fraction is the proportion of new cases that are in the contact networks of recent cases. Therefore the known fraction is entirely determined by how "contact tracing" is implemented: any contact that is not traced is by definition transient. The known fraction is 0.91 for our standard network (see Section A).

In Table 2 we consider three possible values for the known fraction r_s by holding the edges fixed and adjusting the relationship weights. To show power as a function of the known fraction, we fix the trial size to be 100 contact networks of participants. The results show the extent to which power diminishes as the fraction of known transmissions decreases. As contacts become more "known" (thus more contacts are recruited into the trial), the number of confirmed cases within the trial increases, especially in the "null case" where there is no vaccine effect (VE=0). This is a result of the recruitment method better targeting those at risk.

Where it is possible to predict who is at imminent risk of infection, ring recruitment designs are advantageous compared to random recruitment. We expect to see a high known fraction r_s for diseases whose transmission depends on exchange of or exposure to bodily fluids, such as EVD, as well as for COVID-19 in societies under "lockdown", where public spaces are closed and people stay at home, and/or there is extensive quarantining. We expect that the more restrictions to movement and activity there are, the greater the fraction of known transmission events.

Table 2: The effect of the known fraction of transmission events on power. The trial follows the FR design with a follow-up time of 25 days and ends once 100 contact networks have been enrolled. Participants are recruited following the ring strategy. Standard deviations for 10,000 simulations in brackets.

Known fraction r_s	Number of participants	Number of confirmed cases	Vaccinated	Power	Type 1 error	Number of confirmed cases (VE=0)	VE estimate
1.0	2910 (170)	83	1454	0.91	0.04	106	0.59 (0.14)
0.6	2907 (170)	45	1452	0.60	0.05	55	0.55 (0.24)
0.2	2907 (170)	19	1452	0.26	0.05	22	0.43 (0.62)

C.2. Time trend in incidence of infection

Figure 2 shows the robustness of one adaptive trial design to a time trend, demonstrating the effect of the correction of Simon and Simon (2011). We choose to illustrate with linear trends rather than something more realistic, undulating, or stochastic in order to stress test the method. In addition, we choose a trend that we would expect to most favour the Thompson sampling methods. Indeed, we find that uncorrected Thompson sampling sees an increase in type 1 error, which is corrected by the resampling method of Simon and Simon (2011). As TS is a more "aggressive" response-adaptive method than TST, we expect it to be more susceptible to time trends, and therefore the loss in power following correction to be exaggerated.

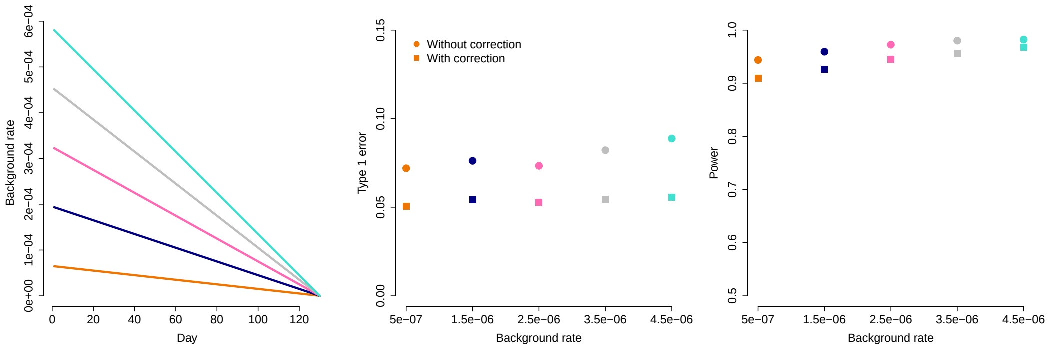

Figure 2: The robustness of one adaptive trial design (TST) to a time trend, demonstrating the effect of the correction of Simon and Simon (2011). Type 1 error rate and power as a function of the trend in the background rate for a trial with a response-adaptive randomisation rate. "Background rate" can be interpreted as the rate of infection by individuals unknown in the context of the trial (e.g. source population or unknown contact). We illustrate with linear trends rather than something more realistic, undulating, or stochastic in order to stress test the method. In addition, we choose a trend that we would expect to most favour the Thompson sampling methods. As TS is a more "aggressive" response-adaptive method than TST, we expect it to be more susceptible to time trends, and therefore the loss in power following correction to be exaggerated. Left: Five different time trends for the background rate. The trend is that background rates diminish over time to zero. Middle: The gradient of the trend is shown on the x axis. On the y axis is the type 1 error rate. Right: The gradient of the trend is shown on the x axis. On the y axis is the power.

C.3. Allocation probabilities

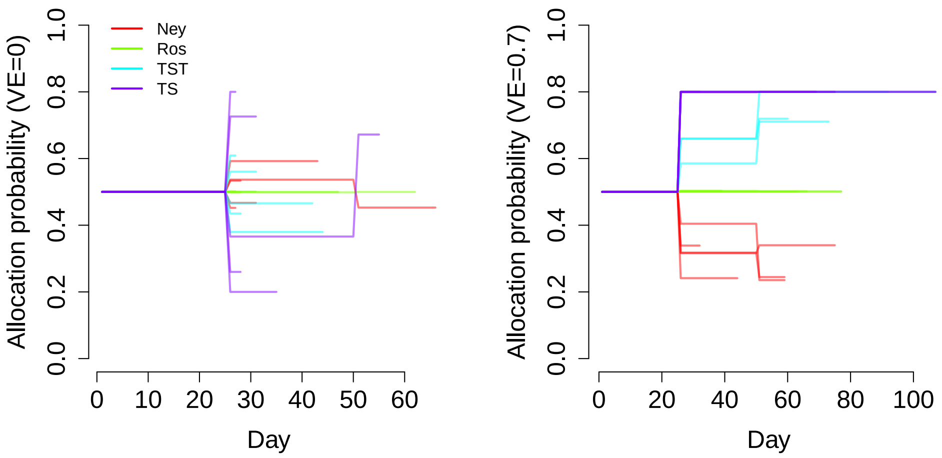

Figure 3: Five samples of trajectories of the allocation probability over time for the adaptive designs, corresponding to Main Text Table 3.

C.4. Thompson sampling methods without early stopping

In Table 3, we show the Thompson sampling methods again, where we do not conclude the trial early for efficacy. This results in much longer trials: on average 6 and 17 days longer (for TST and TS, respectively) than the trials that do stop early, and much harsher power corrections due to the extended periods spent at unequal randomisation, in comparison to Main Text Table 3.

C.5. Fixed-duration trials

We additionally compare the response-adaptive trials when there is a strict limit on their duration and there exists an efficacious vaccine, for example for a trial that lasts at most 85 days, and we evaluate the trial impact in terms of total people vaccinated up to 65 days after the trial's end. First we consider that the same number of people ($N_P = 32$) can be vaccinated per day, and then that ten times as many ($N_P = 320$) can be. Table 4 compares the designs after the total duration of 150 days, where the trial has a fixed duration of at most 85 days, and the number of people vaccinated in the remaining time depends on the probability that the trial successfully identified that the vaccine was efficacious, and the number of people that can be vaccinated per day post-trial. With limited time, the trials that allocate most participants to the efficacious experimental arm – TS and TST

– would be preferable in terms of vaccinating the most people during the trial, and up to day 150, if the rate of vaccination after the trial is the same as the rate of enrolment into the trial. These methods also see fewer exported infection events in the course of the trial – both in total and per day. However, if, after the trial, ten times as many people can be vaccinated per day as were enrolled in the trial, the designs with higher powers are preferable, as they are better able to identify an efficacious vaccine, and therefore will vaccinate more people in the period after the trial.

Table 3: Thompson-sampling response-adaptive designs where the trial design does not include early stopping. The outcome has a continuous weighting. Participants are recruited following the ring strategy. The final follow-up time is 25 days. The trial ends when 24 effective cases have been observed. Standard deviations in brackets. Correction for time trend uses the resampling method of Simon and Simon (2011).

Adaptation	Number of participants	Duration (days)	Number of confirmed cases	Vaccinated	Power	Power (corrected)	Type 1 error	Type 1 error (corrected)	VE estimate	Prevented exported infections
TST	2238 (712)	94 (22)	56	1423	0.76	0.70	0.04	0.04	0.62 (0.19)	6.33
TS	2346 (745)	98 (23)	57	1582	0.74	0.47	0.04	0.06	0.62 (0.19)	6.60

Table 4: Comparison of response-adaptive trials that last at most 85 days. We compare their profiles in terms of the totals vaccinated up to day 150, which includes a maximum of 85 days of vaccinations as part of the trial and a minimum of 65 days of vaccination roll-out given a successful trial. “Vaccinated in trial” is the expected number vaccinated during the trial, and “Vaccinated up to day 150: assuming 32 (320) per day” is the expected number vaccinated after the trial, and up to day 150, estimated as 32 (320) people vaccinated per day, for the days remaining after the end of the trial, multiplied by the power (the probability to have concluded efficacy and rolled out the vaccine — bold text indicates whether uncorrected or drift-corrected power is used). Standard deviations for 10,000 simulations in brackets. Correction for time trend uses the resampling method of Simon and Simon (2011).

								Vaccinated up to day 150:	
Adaptation	Sample size	Duration	Number of confirmed cases	Vaccinated in trial	Power	Power (corrected)	Exported infections in trial[2]	assuming 32 per day	assuming 320 per day
Ney.	1937 (117)	85 (4)	54	808	0.78	**0.73**	24	2,345	16,065
Ros. et al.	1938 (116)	85 (4)	51	976	**0.77**	0.74	24	2,524	16,905
TST	1783 (268)	80 (8)	45	1098	0.73	**0.71**	21	2,696	16,953
TS	1615 (433)	75 (13)	40	997	0.72	**0.64**	20	2,549	16,398
FR	1938 (116)	85 (4)	52	969	**0.77**		24	2,582	16,973

References

Box, G. E. P. (1954). Some theorems on quadratic forms applied in the study of analysis of variance problems, I. Effect of inequality of variance in the one-way classification. *The Annals of Mathematical Statistics*, 25(2):290–302.

European Centre for Disease Prevention and Control (2020). Resource estimation for contact tracing, quarantine and monitoring activities for COVID-19 cases in the EU/EEA. Technical Report March, European Centre For Disease Prevention And Control.

Friede, T. and Kieser, M. (2002). On the inappropriateness of an EM algorithm based procedure for blinded sample size re-estimation. *Statistics in Medicine*, 21(2):165–176.

Fyles, M., Fearon, E., Overton, C., Wingfield, T., Medley, G. F., Hall, I., Pellis, L., and House, T. (2021). Using a household structured branching process to analyse contact tracing in the SARS-CoV-2 pandemic. *medRxiv*, page 2021.02.03.21250992.

Gould, A. L. and Shih, W. J. (1992). Sample size re-estimation without unblinding for normally distributed outcomes with unknown variance. *Communications in Statistics - Theory and Methods*, 21(10):2833–2853.

He, X., Lau, E. H., Wu, P., Deng, X., Wang, J., Hao, X., Lau, Y. C., Wong, J. Y., Guan, Y., Tan, X., Mo, X., Chen, Y., Liao, B., Chen, W., Hu, F., Zhang, Q., Zhong, M., Wu, Y., Zhao, L., Zhang, F., Cowling, B. J., Li, F., and Leung, G. M. (2020). Temporal dynamics in viral shedding and transmissibility of COVID-19. *Nature Medicine*, 26(May).

Henao-Restrepo, A. M., Camacho, A., Longini, I. M., Watson, C. H., Edmunds, W. J., Egger, M., Carroll, M. W., Dean, N. E., Diatta, I., Doumbia, M., Draguez, B., Duraffour, S., Enwere, G., Grais, R., Gunther, S., Gsell, P. S., Hossmann, S., Watle, S. V., Kondé, M. K., Kéïta, S., Kone, S., Kuisma, E., Levine, M. M., Mandal, S., Mauget, T., Norheim, G., Riveros, X., Soumah, A., Trelle, S., Vicari, A. S., Røttingen, J. A., and Kieny, M. P. (2017). Efficacy and effectiveness of an rVSV-vectored vaccine in preventing Ebola virus disease: final results from the Guinea ring vaccination, open-label, cluster-randomised trial (Ebola Ça Suffit!). *The Lancet*, 389(10068):505–518.

Huang, L., Bai, J., Yu, H., and Chen, F. (2018). Sample size re-estimation without un-blinding for time-to-event outcomes in oncology clinical trials. *Journal of Biomedical Research*, 32(1):23–29.

Kiss, I. Z., Miller, J., and Simon, P. (2017). *Mathematics of Epidemics on Networks From Exact to Approximate Models*. Springer.

Kucharski, A. J., Klepac, P., Conlan, A., Kissler, S. M., Tang, M., Fry, H., Gog, J., and Edmunds, J. (2020). Effectiveness of isolation, testing, contact tracing and physical distancing on reducing transmission of SARS-CoV-2 in different settings. *medRxiv*, page 2020.04.23.20077024.

Li, Q., Guan, X., Wu, P., Wang, X., Zhou, L., Tong, Y., Ren, R., Leung, K. S., Lau, E. H., Wong, J. Y., Xing, X., Xiang, N., Wu, Y., Li, C., Chen, Q., Li, D., Liu, T., Zhao, J., Liu, M., Tu, W., Chen, C., Jin, L., Yang, R., Wang, Q., Zhou, S., Wang, R., Liu, H., Luo, Y., Liu, Y., Shao, G., Li, H., Tao, Z., Yang, Y., Deng, Z., Liu, B., Ma, Z., Zhang, Y., Shi, G., Lam, T. T., Wu, J. T., Gao, G. F., Cowling, B. J., Yang, B., Leung, G. M., and Feng, Z. (2020). Early transmission dynamics in Wuhan, China, of novel coronavirus–infected pneumonia. *New England Journal of Medicine*, pages 1–9.

Office for National Statistics (2011). CT0819_2011 Census - household type, household size and age of usual residents (households) - England and Wales.

Simon, R. and Simon, N. R. (2011). Using randomization tests to preserve type I error with response-adaptive and covariate-adaptive randomization. *Statistics Probability Letters*, 81(7):767–772.

Tapiwa, G., Cécile, K., Dongxuan, C., Andrea, T., Christel, F., Jacco, W., and Niel, H. (2020). Estimating the generation interval for COVID-19 based on symptom onset data. *medRxiv*, pages 1–13.

Teel, C., Park, T., and Sampson, A. R. (2015). EM estimation for finite mixture models with known mixture component size. *Commun Stat Simul Comput*, 44(6):1545–1556.
